# Supplementary material for: Dry Needling Alone or in Combination with Exercise Therapy versus Other Interventions for Reducing Pain and Disability in Subacromial Pain Syndrome: A Systematic Review and Meta-Analysis
Source: Int J Environ Res Public Health. 2022 Sep 2;19(17):10961. doi: 10.3390/ijerph191710961 (PMC9518516; doi:10.3390/ijerph191710961)
Supplement: Supplementary file 1 [file ijerph-19-10961-s001.zip › ijerph-1849035-supplementary.pdf]

Supplementary Material:

Table S1. Risk of bias detailed.

**Arias-Buria et al. 2017 [33]**		
Items	Author's judgement	Support for the judgement
Random sequence generation (selection bias)	Low risk	Patients were randomly assigned to experimental or control group using a computer-generated randomized table of numbers.
Allocation concealment (selection bias)	Low risk	Concealed allocation was done by a statistician who did not participate in the main trial. Individual and sequentially numbered index cards with the random assignment were prepared, folded, and placed in sealed opaque envelopes. A second external researcher opened the envelope and proceeded with allocation.
Blinding of participants and personnel (performance bias)	High risk	Not blinded.
Blinding of outcome assessment (detection bias)	High risk	Since outcome measures were self-reported and participants were not blinded, this domain was rated as high risk of bias.
Incomplete outcome data (attrition bias)	Low risk	There were losses during the study (n=1 from the dry needling plus exercise group and n=2 from the exercise group), but data were balanced at the end of intervention and intention-to-treat analysis was used.
Selective reporting (reporting bias)	Low risk	The study protocol was prospectively registered (ClinicalTrials.gov: NCT02338908) and all outcome measures were reported.
Other bias	Low risk	No other sources of bias were detected.
**Ekici et al. 2021 [35]**		
Items	Author's judgement	Support for the judgement
Random sequence generation (selection bias)	Low risk	Randomization was created using a computer-generated randomization table (1:1 ratio).
Allocation concealment (selection bias)	Unclear risk	No information is provided.
Blinding of participants and personnel (performance bias)	High risk	Not blinded.
Blinding of outcome assessment (detection bias)	High risk	Since outcome measures were self-reported and participants were not blinded, this domain was rated as high risk of bias.
Incomplete outcome data (attrition bias)	High risk	There was a substantial incomplete outcome data (n=8 from the dry needling group and n=12 from the deep friction massage group) and per-protocol analysis was used.
Selective reporting (reporting bias)	Unclear risk	No information about the study protocol is provided.
Other bias	Low risk	No other sources of bias were detected.

**Imani et al. 2021 [32]**		
Items	Author's judgement	Support for the judgement
Random sequence generation (selection bias)	Low risk	Participants were randomly placed in three groups using permuted blocked randomization with block size of six and 22 patients were placed in each group.
Allocation concealment (selection bias)	Unclear risk	No information is provided.
Blinding of participants and personnel (performance bias)	High risk	Not blinded.
Blinding of outcome assessment (detection bias)	High risk	Since outcome measures were self-reported and participants were not blinded, this domain was rated as high risk of bias.
Incomplete outcome data (attrition bias)	Low risk	There were losses during the study (n=5 from the dry needling plus physical therapy group, n=1 from the Hong dry needling plus physical therapy group, and n=2 from the physical therapy group), but data were balanced at the end of intervention.
Selective reporting (reporting bias)	Low risk	The study protocol was prospectively registered (IRCT20190409043210N1) and all outcome measures were reported.
Other bias	Low risk	No other sources of bias were detected.

**Jalilipanah et al. 2021 [13]**		
Items	Author's judgement	Support for the judgement
Random sequence generation (selection bias)	Low risk	The selected patients were assigned to the study groups using a permuted block randomization method with block size of 3.
Allocation concealment (selection bias)	Unclear risk	No information is provided.
Blinding of participants and personnel (performance bias)	High risk	Not blinded.
Blinding of outcome assessment (detection bias)	High risk	Since outcome measures were self-reported and participants were not blinded, this domain was rated as high risk of bias.
Incomplete outcome data (attrition bias)	Low risk	There were no losses during the study.
Selective reporting (reporting bias)	Unclear risk	No information about the study protocol is provided.
Other bias	High risk	Four authors have affiliations with organizations with direct or indirect financial interest in the subject matter discussed in the manuscript.

Pérez-Palomares et al. 2017 [34]		
Items	Author's judgement	Support for the judgement
Random sequence generation (selection bias)	Low risk	Participants were assigned to 1 of the 2 groups using a computer-generated random number sequence with no restrictions.
Allocation concealment (selection bias)	Low risk	The information for the random allocation sequence was implemented by phone from an independent researcher, who stated the type of treatment assigned for each new patient. The sequence was concealed throughout the study. Group assignment was carried out by the independent researcher.
Blinding of participants and personnel (performance bias)	High risk	Not blinded.
Blinding of outcome assessment (detection bias)	High risk	Since outcome measures were self-reported and participants were not blinded, this domain was rated as high risk of bias.
Incomplete outcome data (attrition bias)	Low risk	There were losses during the study (n=5 from the dry needling plus physical therapy group and n=6 from the physical therapy group), but data were balanced at the end of intervention and intention-to-treat analysis was used.
Selective reporting (reporting bias)	High risk	There were substantial differences between the study protocol and the manuscript.
Other bias	Low risk	No other sources of bias were detected.

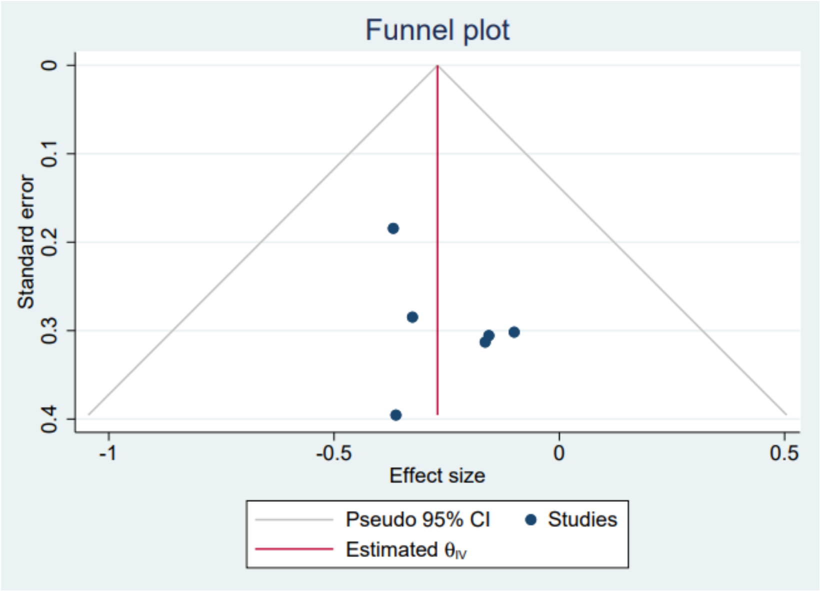

Figure S1. Funnel plot of the meta-analysis of the published studies. Each plotted point represents the effect size (ES) standard error (SE) between post-intervention and pre-intervention Visual Analogue Scale and Numeric Pain Rating Scale in subjects with subacromial syndrome.

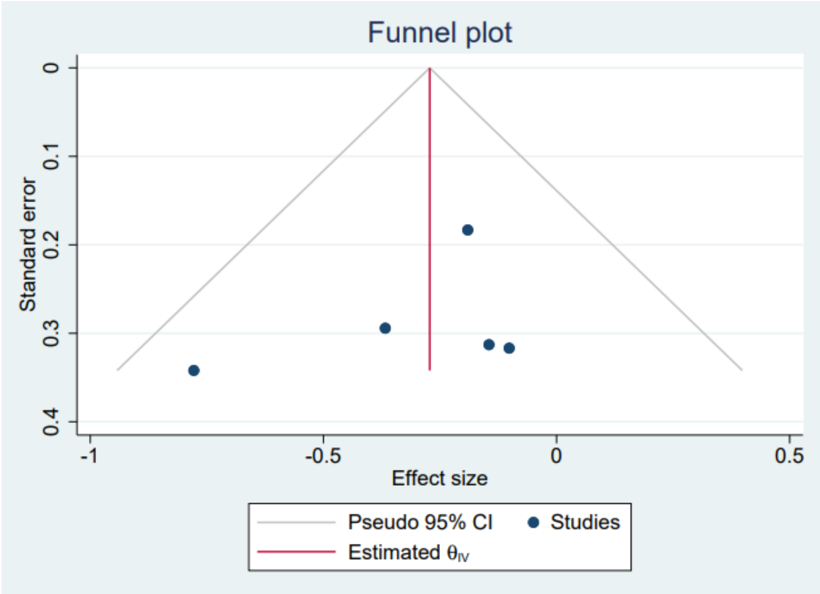

Figure S2. Funnel plot of the meta-analysis of the published studies. Each plotted point represents the effect size (ES) standard error (SE) follow up Visual Analogue Scale and Numeric Pain Rating Scale in subjects with subacromial syndrome.

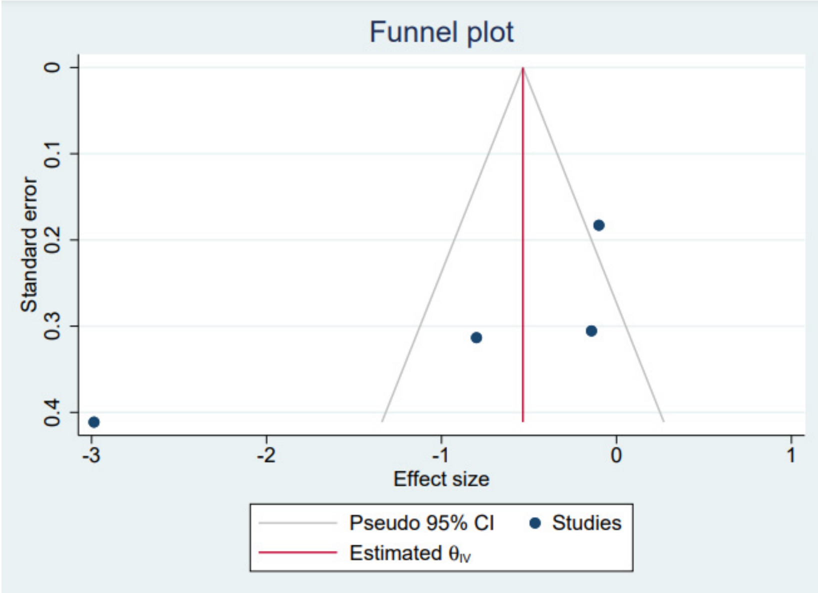

Figure S3. Funnel plot of the meta-analysis of the published studies. Each plotted point represents the effect size (ES) standard error (SE) between post-intervention and pre-intervention Shoulder Pain and Disability Index; Disabilities of the Arm, Shoulder and Hand; and Constant-Murley score in subjects with subacromial syndrome.

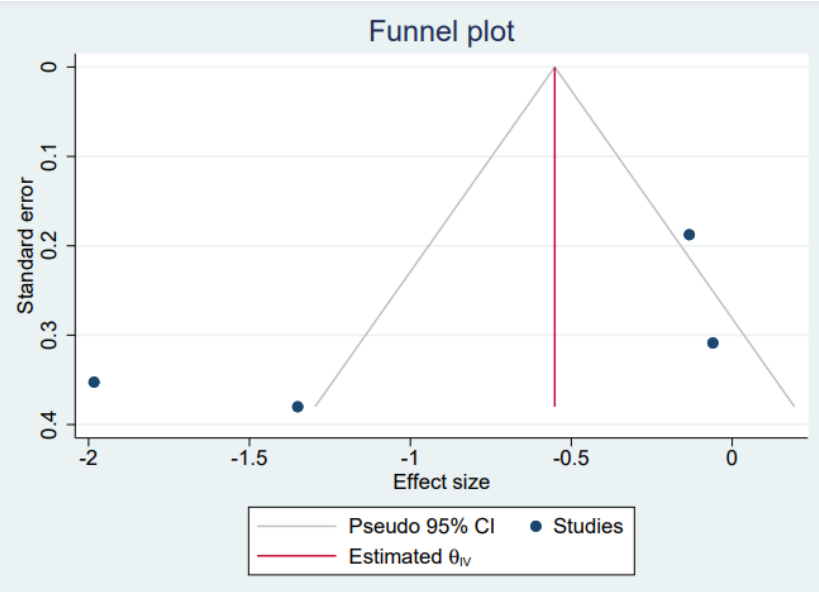

Figure S4. Funnel plot of the meta-analysis of the published studies. Each plotted point represents the effect size (ES) standard error (SE) follow up Shoulder Pain and Disability Index; Disabilities of the Arm, Shoulder and Hand; and Constant-Murley score in subjects with subacromial syndrome.
